# Improved model quality assessment using ProQ2

**DOI:** 10.1186/1471-2105-13-224

**Published:** 2012-09-10

**Authors:** Arjun Ray, Erik Lindahl, Björn Wallner

**Affiliations:** 1 Department of Theoretical Physics & Swedish eScience Research Center, Royal Institute of Technology, Stockholm, Sweden; 2 Center for Biomembrane Research, Department of Biochemistry & Biophysics, Stockholm University, Stockholm, Sweden; 3 Department of Physics, Chemistry and Biology & Swedish eScience Research Center, Linköping University, SE-581 83 Linköping, Sweden

## Abstract

**Background:**

Employing methods to assess the quality of modeled protein structures is now standard practice in bioinformatics. In a broad sense, the techniques can be divided into methods relying on consensus prediction on the one hand, and *single-model* methods on the other. Consensus methods frequently perform very well when there is a clear consensus, but this is not always the case. In particular, they frequently fail in selecting the best possible model in the hard cases (lacking consensus) or in the easy cases where models are very similar. In contrast, single-model methods do not suffer from these drawbacks and could potentially be applied on any protein of interest to assess quality or as a scoring function for sampling-based refinement.

**Results:**

Here, we present a new single-model method, ProQ2, based on ideas from its predecessor, ProQ. ProQ2 is a model quality assessment algorithm that uses support vector machines to predict local as well as global quality of protein models. Improved performance is obtained by combining previously used features with updated structural and predicted features. The most important contribution can be attributed to the use of profile weighting of the residue specific features and the use features averaged over the whole model even though the prediction is still local.

**Conclusions:**

ProQ2 is significantly better than its predecessors at detecting high quality models, improving the sum of Z-scores for the selected first-ranked models by 20% and 32% compared to the second-best single-model method in CASP8 and CASP9, respectively. The absolute quality assessment of the models at both local and global level is also improved. The Pearson’s correlation between the correct and local predicted score is improved from 0.59 to 0.70 on CASP8 and from 0.62 to 0.68 on CASP9; for global score to the correct GDT_TS from 0.75 to 0.80 and from 0.77 to 0.80 again compared to the second-best single methods in CASP8 and CASP9, respectively. ProQ2 is available at http://proq2.wallnerlab.org.

## Background

Modeling of protein structure is a central challenge in structural bioinformatics, and holds the promise not only to identify classes of structure, but also to provide detailed information about the specific structure and biological function of molecules. This is critically important to guide and understand experimental studies: It enables prediction of binding, simulation, and design for a huge set of proteins whose structures have not yet been determined experimentally (or cannot be obtained), and it is a central part of contemporary drug development.

The accuracy of protein structure prediction has increased tremendously over the last decade, and today it is frequently possible to build models with 2-3Å resolution even when there are only distantly related templates available. However, as protein structure prediction has matured and become common in applications, the biggest challenge is typically not the overall average accuracy of a prediction method, but rather how accurate a specific model of a specific protein is. –Is it worth spending months of additional human work, modeling and simulation time on this model? Ranking or scoring of models has long been used to select the best predictions in methods, but this challenge means there is also a direct need for *absolute* quality prediction, e.g. the probability of a certain region of the protein being within 3Å of a correct structure.

One of the most common prediction approaches in use today is to produce many alternative models, either from different alignments and templates [1-4] or by sampling different regions of the conformational space [5]. Given this set of models, some kind of scoring function is then used to rank the different models based on their structural properties. Ideally, this scoring function should correlate perfectly with the distance from the native structure. In practice, while they have improved, ranking methods are still not able to consistently place the best models at the top. In fact, it is often the case that models of higher or even much higher quality than the one selected are already available in the set of predictions, but simply missed [6,7]. In other words, many prediction methods are able to generate quite good models, but we are not yet able to identify them as such! In principle, there are three classes of functions to score protein models. The first of them is *single-model methods* that only use information from the actual model, such as evolutionary information [8-10], residue environment compatibility [11], statistical potentials from physics [12] or knowledge-based ones [13,14], or combinations of different structural features [15-19]. The second class is *consensus methods* that primarily use consensus of multiple models [1] or template alignments [20] for a given sequence to pick the most probable model. Finally, there are also *hybrid methods* that combine the single-model and consensus approaches to achieve improved performance [21-24]. Of the above methods, it is only the single-model methods that can be used for conformational sampling and as a guide for refinement since they are strict functions of the atomic positions in the model. On the other hand, in terms of accuracy the consensus and hybrid methods outperform the single methods, in particular in benchmarks such as CASP [25] with access to many alternative models for all different targets. The success of the consensus methods in CASP has resulted in an almost complete lack of development of new true single-model methods. As a consequence only 5 out of 22 methods submitting predictions to both the global and local categories in the model quality assessment part of the latest CASP were actual true single-model methods [25]. By *true*, we mean methods that can be used for conformational sampling and that do not use any template information in the scoring of models.

Scoring of models can be performed at different levels, either locally (i.e., per residue) or globally to reflect the overall properties of a model. Traditionally, the focus of most scoring functions has been to discriminate between globally incorrect and approximately correct models, which works reasonably well e.g. for picking the model that provides the best average structure for a complete protein (which is highly relevant e.g. for CASP). In contrast, only a handful of methods focus on predicting the individual correctness of different parts of a protein model [9,11,23,26], but this is gradually changing with the introduction of a separate category for local quality assessment in CASP. In fact, we believe that local quality prediction might even be more useful than global prediction. First, it is relatively easy to produce a global score from the local, making global scoring a special case of the local one. Second, a local score can be used as a guide for further local improvement and refinement of a model. Third, even without refinement local quality estimates are useful for biologist as it provides confidence measures for different parts of protein models.

In this study, we present the development of the next generation of the ProQ quality prediction algorithm, and how we have been able to improve local quality prediction quite significantly through better use of evolutionary information and combination of locally and globally predicted structural features. ProQ was one of the first methods that utilized protein models, in contrast to native structures, to derive and combine different types of features that better recognize correct models [15]. This was later extended to local prediction in ProQres [9] and to membrane proteins [27]. We have reworked the method from scratch by using a support vector machine (SVM) for prediction, and it has been trained on a large set of structural models from CASP7. In addition to evolutionary data, there are several new model features used to improve performance significantly, e.g. predicted surface area and a much-improved description of predicted secondary structure. We also show that including features averaged over the entire model, e.g. overall agreement with secondary structure and predicted surfaces, improves local prediction performance too.

## Results and discussion

The aim of this study was to develop an improved version of ProQ that predict local as well as global structural model correctness. The main idea is to calculate scalar features from each protein model based on properties that can be derived from its sequence (e.g. conservation, predicted secondary structure, and exposure) or 3D coordinates (e.g. atom-atom contacts, residue–residue contacts, and secondary structure) and use these features to predict model correctness (see Methods for a description of the features new to this study). To achieve a localized prediction, the environment around each residue is described by calculating the features for a sliding window centered around the residue of interest. For features involving spatial contacts, residues or atoms outside the window that are in spatial proximity of those in the window are included as well. After the local prediction is performed, global prediction is achieved by summing the local predictions and normalize by the target sequence length to enable comparisons between proteins. Thus, the global score is a number in the range [0,1]. The local prediction is the local S-score, as defined in the Methods section.

### Development of ProQ2

From the earlier studies, we expect optimal performance by combining different types of input features [15,17,18]. To get an idea of which features contribute most to the performance, support vector machines (SVMs) were trained using five-fold cross–validation on individual input features as well as in combination of different feature types. After trying different SVM kernels (including linear, radial basis function and polynomial ones), we chose the linear kernel function for its performance, speed and simplicity.

The Pearson’s correlation coefficient for SVMs trained with different input features is shown in Table 1. First, we retrained ProQ on CASP7. The original version of ProQ used neural networks and as expected the performance did not change much merely with the change of machine learning algorithm. The difference is well within what would be expected by chance. This retrained version of ProQ was used as the baseline predictor against which new single features were tested. In this way, any improvement over ProQ will easily be identified as significant improvements over the baseline.

**Table 1:** Pearson’s correlation coefficient for different input features

**Training data**	**Pearson’s correlation**
**ProQ**	**0.54 (±0.006)**
**Retrained ProQ (Base)**	**0.55 (±0.006)**
Atom	0.43 (±0.006)
Residue	0.27 (±0.008)
Surface	0.47 (±0.006)
Residue + Profile Weighting	0.32 (±0.007)
Surface + Profile Weighting	0.51 (±0.006)
Base + Global Surface Area Prediction	0.65 (±0.005)
Base + Global Secondary Structure Pred.	0.65 (±0.005)
Base + Profile Weighting	0.62 (±0.005)
Base + Local Surface Area Prediction	0.58 (±0.005)
Base + Local Secondary Structure Pred.	0.58 (±0.005)
Base + Information per position (Conservation)	0.56 (±0.006)
All Combined (ProQ2)	0.71 (±0.004)

Overall Pearson’s correlation coefficient, for different input features, benchmarked using cross-validation on the CASP7 data set. (Errors correspond to 99.9% confidence intervals).

The largest performance increase in local prediction accuracy is actually obtained by including global features describing the agreement with predicted and actual secondary structure, and predicted and actual residue surface area calculated as an average over the whole model. Even though these features are not providing any localized information, they increase the correlation between local predicted and true quality significantly over the baseline (+0.10 to 0.65). The performance increase is about the same for predicted secondary structure and predicted surface area. The use of *global* features, i.e. features calculated over the whole model to predict *local* quality, is not as strange as it first might seem. The global features reveal whether the model is overall accurate, an obvious prerequisite for the local quality to be accurate. For instance, from the local perspective a model might appear correct, i.e. favorable interactions and good local agreement with secondary structure prediction, but a worse global agreement could affect the the accuracy in the first region too. Both predicted secondary structure and predicted surface area are also among the local features that result in a slight performance increase (+0.03 to 0.58).

The second-largest performance increase is obtained by *profile weighting* (+0.07 to 0.62). This is actually not a new feature, but rather a re-weighting of the residue-residue contact and surface area features used in the original version of ProQ, which is here based on multiple sequence alignment of homologous sequences. This re-weighting improves the performance of residue-residue contacts and surface area based predictors to equal degree (Table 1).

Finally, a small increase is also observed by adding the information per position from the PSSM, a measure of local sequence conservation. This is despite the fact that this type of information in principle should have been captured by the feature describing correspondence between calculated surface exposure and the one predicted from sequence conservation.

### Combining ProQ2 with Pcons

It has been shown many times, both in CASP [25,28,29] and elsewhere [1,2,21], that consensus methods are superior MQAPs compared to stand-alone or single methods not using consensus or template information, at least in terms of correlation. However, a major drawback with consensus methods is that they perform optimally in the fold recognition regime, but tend to do worse in free modeling where consensus is lacking or for easy comparative modeling targets, where consensus is all there is. Even though the correlation can be quite high, they often fail in selecting the best possible model.

**Figure 1:**
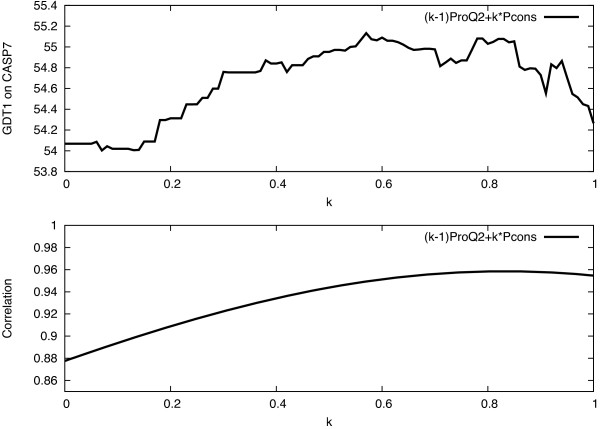
Optimization of linear combination of ProQ2 and Pcons to improve model selection.

Here, we combine the structural evaluation made by ProQ2 with consensus information from Pcons to overcome some of the problems with model selection for consensus based methods. The ProQ2 and Pcons scores are combined using a linear sum with one free parameter:

$$S_{\text{ProQ2+Pcons}}=(1-k)S_{\text{ProQ2}}+k\cdot S_{\text{Pcons}}\;k\in [0,1],$$

where *k* was optimized to *k*=0.8 to maximize GDT1 (Figure 1). Other ways to combine the two scores were tried but this linear combination showed the best performance. Since both the ProQ2 and the Pcons score reflect model correctness, a linear combination makes sense. In the case of free-modeling targets the consensus score will be low and most of the selection will be made on the ProQ2 score. Analogously, in the case of easy comparative modeling targets the consensus score will be high but it will be high for most of the models, and the selection will again essentially be done by the ProQ2 score.

Overall for CASP7 targets, the combination selects models that are of 1.4% and 1.8% higher quality compared to ProQ2 and Pcons respectively, while maintaining a good correlation. The bootstrap support values calculated according to [30], with repeated random selection with return, are higher than 0.95, which demonstrates that GDT1 for the combination is higher in more than 95% of the cases.

### Benchmark of local model correctness

For the benchmarking of model correctness, both at the local and global level, a set of models from CASP8 and CASP9 was used. Since ProQ2 was trained on CASP7, this set is completely independent. To be able to compare the performance, predictions from top-performing MQAPs were also included in the benchmark (Table 2). Unfortunately, not all of these methods had predictions for all models and all residues, so we filtered the number of models to a set for which there were predictions from all methods. Even though this excluded many models, there were still more than 8.2 million residues from over 42,000 models remaining.

**Table 2:** Description of the methods included in the benchmark

**Method**	**Description**
**ProQ2** (S)	**Support Vector Machine trained to predict S-score**
ProQ^∗^ (S)	Neural network trained on structural features to predict LGscore [15] and S-score [9].
QMEAN (S)	Potential of mean force, top-ranked single MQAP in CASP8 and CASP9 [18]
MetaMQAP (S)	Neural network trained on the output from primary MQAPs [16]
Distill_NNPIF (S)	Neural network trained on CA-CA interactions [25]
ConQuass (S)	Correlates conservation and solvent accessibility, only global [10]
MULTICOM-CMFR (S)	Top-ranked single MQAP in CASP8, only global [17].
QMEANclust (C)	QMEAN-weighted GDT_TS averaging, top-ranked consensus method MQAP in CASP8 and CASP9 [23].
**ProQ2+Pcons** (C)	**Linear combination of ProQ2 and Pcons scores, 0.2ProQ2+0.8Pcons**

Description of the single-model methods and the reference consensus method included in the benchmark. The single methods (S) do not use any template or consensus information. Consensus and hybrid methods (C) are free to use any type of information. ^∗^This method was originally called ProQres, but for clarity it will be referred to as ProQ both for global and local quality prediction. (S) single-model method (C) consensus method.

We focused the benchmark on different properties that cover various aspects of local model correctness. The most obvious is the correspondence between predicted and true local quality score, which was measured by correlation at different levels (Table 3). The performance of the single-model methods is similar on both CASP8 and CASP9 data sets. Among all the methods, ProQ2 correlates best with the correct local S-score, achieving correlations of 0.70 and 0.68 for CASP8 and CASP9, respectively. The improvement over the second-best MQAP in CASP9 (MetaMQAP, R=0.62) is significant with P-value <10^−127^ using the Fisher R to z transform (Table 4).

**Table 3:** Local model quality benchmark on the CASP8/CASP9 data sets

**Method**	**R**	**〈*R***_***target***_**〉**	**〈*R***_***model***_**〉**
ProQ2	0.70/0.68	0.58/0.54	0.54/0.47
MetaMQAP	–/0.62	–/0.48	–/0.42
QMEAN	0.59/0.59	0.51/0.49	0.49/0.44
ProQ	0.52/0.49	0.46/0.42	0.45/0.40
QMEANclust	0.83/0.77	0.73/0.70	0.68/0.61

Benchmark of local model quality on the CASP8/CASP9 data sets measured by correlations. *R* is overall Pearson’s correlation, 〈*R*_*target*_〉 is the average correlation per target, 〈*R*_*model*_〉 is the average correlation per model. First value correspond to CASP8, second to CASP9. The standard error is <0.002.

**Table 4:** Statistical significance test for local quality prediction

**Method**		**1**	**2**	**3**	**4**	**5**
ProQ2	1		-127.47	-142.98	-170.37	-152.64
MetaMQAP	2	n/a		-95.06	-152.15	-170.69
QMEAN	3	-146.99	n/a		-140.19	-176.87
ProQ	4	-164.75	n/a	-121.07		-191.26
QMEANclust	5	-168.81	n/a	-188.12	-195.66	

Pairwise statistical significance test on the correlation coefficients for local quality prediction from CASP8 (below diagonal) and CASP9 (above diagonal). The values correspond to the logarithm of the P-value for methods being statistically indistinguishable, obtained by comparing the distribution of Fisher’s Z (Eq: 1) for the correlation coefficients, *R*, from Table 3.

**Figure 2:**
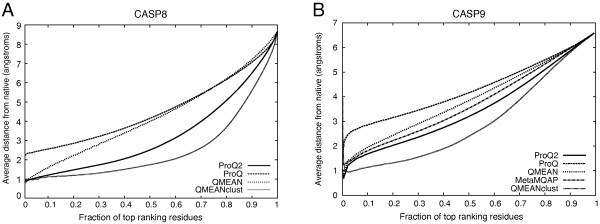
Local quality prediction performance as measured by the average distance deviation for different fraction of top ranking residues for CASP8 (A) and CASP9 (B).

To get an idea of how good the top-ranking residues from the different methods are, we also calculated the average distance deviations from the true value for different fraction of top-ranking residues (Figure 2). This measure should ideally be as low as possible, but will gradually increase to the average deviation over the whole set. On CASP8 (Figure 2A), ProQ2 has a much lower average distance than ProQ and lower compared to QMEAN for the same level of top ranking residues. This is also maintained on the CASP9 data set, even though the distance to QMEAN is smaller and the new single-model method MetaMQAP performs between ProQ2 and QMEAN.

**Figure 3:**
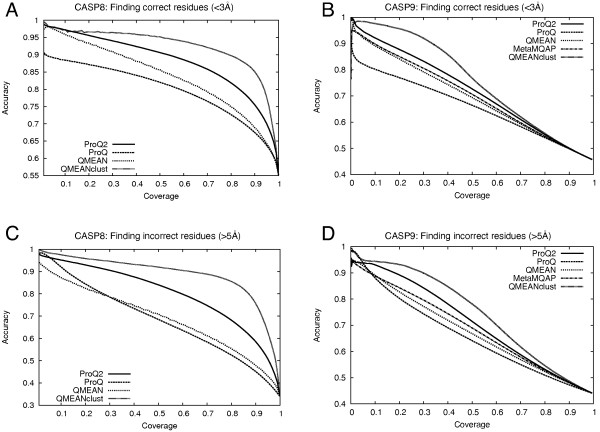
Accuracy for finding correct and incorrect residues for different coverage levels on CASP8 (A) and CASP9 (B).

Finally, the ability to recognize correct and incorrect residues was analyzed using receiver operating characteristic (ROC) plots with cutoffs of <3Å and >5Å deviations for correct and incorrect residues, respectively (Figure 3). For clarity, we have excluded our local consensus servers, Pcons and the ProQ2+Pcons combination, in all figures and only include the top-ranked consensus method from CASP8, QMEANclust, as comparison. All consensus methods perform similarly at least in comparison to single-model methods (data not shown). The relative ranking of the methods based on the ability to recognize correct and incorrect residues (Figure 3) is the same as using the average distance deviation (Figure 2). However, ProQ2 seem to be relatively better at finding incorrect residues.

In all of the above tests the performance of the reference consensus method, QMEANclust, is of course better than any of the single-model methods, but the performance gap is now significantly smaller.

### Benchmark of global model correctness

In the benchmark of global model correctness, the ability to predict global quality as well as the ability to select models was assessed. The first ability is important for assessing how reliable the predicted quality estimates are. This is not necessarily the same as the model selection ability, which only is about selecting the best possible model. The result from the global benchmark is shown in Table 5. The quality predicted by ProQ2 has a correlation coefficient of 0.80 on both CASP8 and CASP9 data sets. This is significantly better (*P* < 10^−27^, and *P* < 10^−14^, respectively) than the correlation for single-model method with second highest correlation, QMEAN (R=0.75/0.77) (Table 6). In fact all correlation differences except QMEAN and MetaMQAP on CASP9 are statistical significant at the 0.001 significance level.

**Table 5:** Benchmark of global model quality

**Single-model methods**	**R**	**R**_***target***_	**∑GDT1**	$\sum Z_{\text{GDT1}}$
**ProQ2**	0.80/0.80	0.72/0.69	75.2/47.0	100.4/68.6
QMEAN	0.75/0.77	0.71/0.66	73.6/44.7	81.1/52.1
MetaMQAP	–/0.76	–/0.59	–/43.1	–/40.3
ConQuass	–/0.73	–/0.66	–/40.4	–/20.4
Distill_NNPIF	–/0.71	–/0.64	–/43.9	–/43.5
MULTICOM-CMFR	0.71/–	0.68/–	74.0/–	83.7/–
ProQ	0.67/0.68	0.65/0.54	71.5/42.3	59.3/40.0
**Consensus methods**				
QMEANclust	0.89/0.96	0.94/0.91	75.8/48.6	104.1/81.41
MULTICOM-CLUSTER	0.96	0.91	48.7	82.3
Mufold	0.96	0.91	48.7	82.5
**ProQ2+Pcons**	0.89/0.95	0.94/0.89	76.9/48.7	118.5/81.6
Pcons	0.89/0.95	0.95/0.91	75.9/48.3	101.6/76.8
PconsM	0.95	0.90	47.9	70.2
United3D	0.95	0.92	48.8	81.2
MUFOLD-QA	0.95	0.92	48.3	79.5
ModFOLDclust2	0.95	0.90	48.4	80.6
MetaMQAPclust	0.95	0.91	48.4	78.2
IntFOLD-QA	0.95	0.90	48.4	79.9
MULTICOM-REFINE	0.94	0.88	46.2	66.6
MULTICOM	0.94	0.88	48.7	84.7
MQAPmulti	0.94	0.91	48.2	75.4
ModFOLDclustQ	0.94	0.87	48.6	82.3
MQAPsingle	0.92	0.81	45.3	45.2
MULTICOM-CONSTRUCT	0.90	0.82	46.6	63.3
gws	0.90	0.81	45.3	44.2
Splicer	0.89	0.85	47.6	75.4
LEE	0.89	0.80	45.1	42.9
Splicer_QA	0.88	0.84	47.8	77.4
Modcheck-J2	0.87	0.77	41.7	26.2
MUFOLD-WQA	0.86	0.91	49.0	83.9
SMEG-CCP	0.83	0.76	47.9	74.9
QMEANdist	0.80	0.84	47.8	77.1
QMEANfamily	0.75	0.68	44.8	50.3
GRIER-CONSENSUS	0.68	0.86	48.3	82.0
Baltymus	0.58	0.53	41.8	32.8
**Best Possible**	1.00/1.00	1.00/1.00	82.3/52.2	182.2/127.9

Benchmark of global model quality on CASP8 and CASP9 data sets. *R* is overall correlation, R_*target*_ the average correlation per target, ∑GDT1 is the the sum of the first-ranked models for each target and ∑Z_*GDT1*_ is the summed Z-score for the first-ranked models for each target. The first value corresponds to results on CASP8, the second to CASP9, and cells with only one value are CASP9 only.

**Table 6:** Statistical significance test for global quality prediction

**Method**		**1**	**2**	**3**	**4**	**5**	**6**	**7**	**8**
ProQ2	1		-14.48	-21.73	-56.80	-39.07	-47.35	n/a	-103.07
QMEAN	2	-29.86		−1.91	-43.51	-18.50	-30.30	n/a	-106.70
MetaMQAP	3	n/a	n/a		-38.46	-11.29	-23.67	n/a	-107.76
ProQ	4	-61.20	-39.03	n/a		-20.91	-8.69	n/a	-114.42
ConQuass	5	n/a	n/a	n/a	n/a		-4.79	n/a	-110.58
Distill_NNPIF	6	n/a	n/a	n/a	n/a	n/a		n/a	-112.22
MULTICOM-CMFR	7	-49.09	-17.94	n/a	-14.99	n/a	n/a		n/a
QMEANclust	8	-65.82	-79.03	n/a	-91.56	n/a	n/a	-86.12	

Pairwise statistical significance test on the correlation coefficients for global quality prediction from CASP8 (below diagonal) and CASP9 (above diagonal). The values correspond to the logarithm of the P-value for methods being statistically indistinguishable, obtained by comparing the distribution of Fisher’s Z (Eq: 1) for the correlation coefficients, *R*, from Table 5. Values in italics are not distinguishable at the 10^−3^significance level.

The model selection as measured by the sum of GDT_TS for the first ranked model is also significantly improved, from 74.0/44.7 for the best single-model methods in CASP8 (MULTICOM-CMFR) and CASP9 (QMEAN) to 75.2/47.0 for ProQ2 on the two data sets. To put the numbers in perspective, the performance is now closer to the selection of the reference consensus method QMEANclust (75.8/48.6) than to the selection of the previous best performing single-model methods. In addition, when ProQ2 is combined with our consensus method Pcons, the model selection is improved further to 76.9/48.7. This performance is similar to the best performing consensus methods and clearly better than Pcons alone (75.9/48.3), which demonstrates the added value of ProQ2.

**Figure 4:**
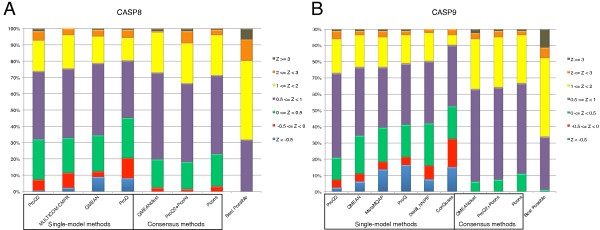
Distribution of Z-score for model selection on CASP8 (A) and CASP9 (B).

Although it is commonly used as a measure of model selection, the sum of GDT_TS for the first ranked model gives higher emphasis to easier targets, since these will have higher GDT_TS. To analyze the target selection in more detail, we calculated Z-scores by subtracting the mean quality from the quality of the selected model and dividing by the standard deviation for each target. These Z-scores are not biased by target difficulty, since the scores are normalized by the quality distribution for each target. Thereby it also directly measures the added value of the model quality assessment program over a random pick, which would have a Z-score of zero. The distributions of Z-scores for the different methods are shown in Figure 4.

ProQ2 has lower number of predictions below average (Z<0) and larger number of predictions with Z-score greater than 2 compared to the other single-model methods. Only for a few targets (3/203 on CASP8 and CASP9) does the model selected by ProQ2 have a Z<-0.5, while both QMEAN and ProQ have more than 15 models with Z-scores in that range. This is in sharp contrast to the consensus methods that never select models with Z<-0.5 and on CASP9 none of the consensus methods select models with Z<0, demonstrating that consensus methods seldom select models worse than average. This is one of the clear advantages over non-consensus methods. However, at the other end of the spectrum, the ability of the consensus method to select models with high Z-score is quite far from optimal, as demonstrated by the Z-score distribution for Pcons and QMEANclust on the CASP8 data set in Figure 4A. In fact, all single-models methods select more models with Z>2 than either Pcons or QMEANclust, indicating that combining the results from single methods with consensus could potentially improve the results at the high end. This is also exactly what is observed for ProQ2+Pcons, were the number of models with Z>2 increases significantly from 8 for Pcons to 15 for ProQ2+Pcons on the combined CASP8/CASP9 set (bootstrap value:>0.92).

## Conclusions

The aim of this study was to improve both local and global *single-model* quality assessment. This was done by training support vector machines to predict the local quality measure, S-score, with a combination of evolutionary and multiple sequence alignment information combined with other structural features on data from CASP7. The final version, ProQ2, was compared to the top-performing single-model quality assessment groups in CASP9 as well as to its predecessor, ProQ, on the complete CASP9 data set.

We show that ProQ2 is superior to ProQ in all aspects. The correlation between predicted and true quality is improved from 0.52/0.49 (CASP8/CASP9) to 0.70/0.68 on the local level and from 0.67/0.68 to 0.80/0.80 on the global level. In addition, the selection of high-quality models is also dramatically improved. ProQ2 is significantly better than the top-performing single-model quality assessment groups in both CASP8 and CASP9. The improvement in correlation is larger on the local level, but still significant on the global level. The largest improvement is however in the selection of high-quality models with a sum of Z-score improvement from 83.7/52.1 on CASP8/CASP9 for the second-ranked single model to 100.4/68.6 for ProQ2. Finally, we also show that ProQ2 combined with the consensus predictor Pcons can improve the selection even further.

## Methods

### Test and training data

The performance from any machine learning approach is ultimately limited by the quality of the underlying data used for training and testing. For the development of ProQ2 we used data from CASP7 [31] for training and data from CASP8 [6] and CASP9 [7] for testing and benchmarking. Each residue in each protein model generates one input item, so using the complete CASP7 data set would have resulted in far too much training data. Instead, we selected ten representative models from each target randomly, resulting a final set of 163,934 residues from 874 models of 102 target structures.

The testing and benchmarking data from CASP8 and CASP9 included all models and the top-ranked model quality assessment programs from the MQAP category in CASP. ProQ2 participated officially in CASP9 so those predictions were truly on unseen data. To make the present comparison fair, we had to filter out all models that did not have a prediction for all targets included in the benchmark. This resulted in a final benchmarking set consisting of 3,620,156 residues from 18,693 models from 122 targets for CASP8 and 4,676,538 residues from 23,635 models from 81 targets and 21,589 models from 81 targets for the local and global quality benchmark for CASP9, respectively. The reason for the different number of models is that we wanted to include as many global single-model methods as possible (there were fewer methods making local predictions), thereby making the union of a common set smaller than for the local.

### SVM training

SVM training was performed using five-fold cross-validation on the CASP7 data set. The SVM_*light*_[32] V6.01 implementation of support vector machine regression was used with a linear kernel function (other kernels were tried but showed no increased performance). The trade-off between training error and margin was optimized (the -c parameter) and the epsilon for the width-of-loss function in regression tube was optimized for all cross-validation sets at the same time.

### Training parameters

Support vector machines were trained using structural features describing the local environment around each residue in the protein models combined with other features predicted from sequence such as secondary structure, surface exposure and conservation.

Combinations of the following features were used: atom–atom and residue–residue contacts, surface accessibility, predicted secondary structure, predicted surface exposure, and evolutionary information calculated over a sequence window. Many of the features, in particular the structural ones, are similar to those used in our earlier studies [9,15]. For consistency we include a short description of them here.

#### Atom–atom contacts

This features describes the distribution of atom–atom contacts in the protein model. Atoms were grouped into 13 different atom types based on chemical properties, see Wallner *et al.* (2003). Two atoms were defined to be in contact if the distance between them was within 4Å. The 4Å cutoff was chosen by trying different cutoffs in the range 3Å–7Å. Contacts between atoms from positions adjacent in sequence were ignored. Finally, the number of contacts from each group was normalized by dividing with the total number of contacts within the window.

#### Residue–residue contacts

This feature describes the distribution of residue–residue contacts. Residues were grouped in six different groups: (1) Arg, Lys; (2) Asp, Glu; (3) His, Phe, Trp, Tyr; (4) Asn, Gln, Ser, Thr; (5) Ala, Ile, Leu, Met, Val, Cys; (6) Gly, Pro [15,33]. Two residues were defined to be in contact if the distance between the C*α*–atoms or any of the atoms belonging to the side chain of the two residues were within 6Å and if the residues were more than five residues apart in sequence. Many different cutoffs in the range 3Å–12Å were tested and 6Å showed the best performance. Finally, the number of contacts for each residue group was normalized with the total number of contacts within the window.

#### Solvent accessibility surfaces

This features describes the exposure distribution for the same residue grouping as used for the residue–residue contacts. The surface accessibility was calculated using NACCESS [34]. The relative exposure of the side chains for each residue group was used. The exposure data was grouped into one of the four groups <25%, 25%–50%, 50%–75% and >75% exposed and finally normalized by the number of residues within the window.

#### Secondary structure

This set of features describes the secondary structure in the model and how it corresponds to the secondary structure predicted from the sequence. STRIDE [35] was used to assign one of three secondary structure classes (helix, sheet, or coil) to each residue in the protein models based on coordinates. PSIPRED [36] was used to predict the probability for the same secondary structure classes. From these, three sets of features were calculated: 

1. The predicted probability from PSIPRED for the secondary structure of the central residue in the sequence window.

2. Correspondence between predicted and actual secondary structure over a 21-residue window.

3. Secondary structure assigned by STRIDE, binary encoded into three classes over a 5-residue window.

We also tried secondary structure content for the three classes over a sequence window but that did not show any improved prediction performance.

#### Surface area

This set of features describes the surface area in the model and how it corresponds to predicted surface area. The accessible residue surface area was calculated with NACCESS [34] and residues exposing <25% of the side-chain were classified as buried while the others were classified as exposed. The residue burial and exposure was also predicted by ACCpro [37]. From these classifications and predictions we calculated: 

1. Correspondence between predicted and actual burial/exposure class over a 21-residue window.

2. Actual surface area over a 13-residue window. This feature complements the surface features that describe the exposure pattern for different residues used earlier.

#### Evolutionary information

This describes the evolutionary history of a given sequence window. Sequence profiles were derived using three iterations of PSI-BLAST [38] against UniRef90, release 2010_3 [39] with a 10^−3^ E–value cutoff for inclusion (-h) and all other parameters at default settings. The sequence profile output from PSI-BLAST contains both the position-specific scoring matrix (PSSM) and the ’information per position’ (IPP), a position-specific measure of conservation calculated from the PSSM. The PSSM was used for weighting of the structural features (see “Profile weighting” section below). The IPP was used directly as an input feature with an optimized window size of 3.

#### Profile weighting

This is actually not an individual feature but rather a re-weighting of all residue-based features, i.e the residue-residue contacts and residue-specific exposure patterns, according to the occurrence in the sequence profile. For instance, if a position in the sequence profile contains 40% alanine and 60% serine, contacts to this position are weighted by 40% as contacts alanine and by 60% as contacts to serine. This effectively increases the amount of training examples and should also make the final predictor less sensitive to small sequence changes, since data is extracted from multiple sequence alignments among homologous sequences.

#### Global features

All the features described above are localized to a short window in sequence, to enable a localized quality prediction. However, it turned out that including the following global features, i.e features calculated over the whole model instead of a window, improved performance significantly even for the local quality prediction: 

1. Global correspondence between predicted and actual secondary structure.

2. Global correspondence between predicted and actual residue burial/exposure.

### Target function

In this study, we have used S-score as the correctness measure for each residue in a protein model. This score was originally developed by Levitt *et al.* (1998) and is defined as:

$$S_{i}=\frac{1}{1+{\left(d_{i}/d_{0}\right)}^{2}}$$

where *d*_*i*_is the distance for residue *i* between the native structure and the model and *d*_0_is a distance threshold. This score ranges from 1 for a perfect prediction (*d*_*i*_ = 0) to 0 when *d*_*i*_goes to infinity. The distance threshold defines the distance at which the score should be 0.5 and it controls how fast the function should go to zero. Here, the distance threshold was set to 3Å and *S*_*i*_ was calculated from the superposition that gave the highest sum of *S*_*i*_over the whole model, in the same way as in MaxSub [40]. In addition, for benchmarking purposes we have also used GDT_TS [41], which is the CASP-standard. It is sometimes more intuitive for non-experts to express the S-score as a distance deviation, solving the above equation for *d*_*i*_:

$$\begin{array}{l} d_{i}=d_{0}\sqrt{1/S_{i}-1}=\{d_{0}=3\text{Å},S_{i}\in (0,1]\}\\ \;=3\sqrt{1/S_{i}-1}\;\text{Å} \end{array}$$

### Performance measures

It is important to employ performance measures that reflect predictive capabilities. Ideally, a performance measure should be a single score that can be used to rank different predictions. Throughout this study, Pearson’s correlation, *R*, is used to rank both local and global predictions. To compare two correlations, Fisher’s *R* to *z* transformation is used:

(1) $$z=0.5\left[\text{ln}(1+R)-\text{ln}(1-R)\right],$$

where *R* is Pearson’s correlation and *z* a normally distributed variable with variance *s*^2^=1/(*n*−3), with *n* being the number of observations. Two correlation coefficients *R*_1_ and *R*_2_ can be converted into the corresponding *z*_1_and *z*_2_. The P-value associated with |*z*_1_−*z*_2_| calculated from the normal distribution is an estimate of the likelihood that the difference between *R*_1_ and *R*_2_ is significant. Fisher’s transformation is used both for P-value estimates, as well as for constructing confidence intervals.

For benchmarking global quality, an additional measure called GDT1 was also used. This measure is simply the sum of the GDT_TS score for the highest-ranked model by the different methods for each target, and it is commonly used in CASP. In contrast to the correlation coefficient this measure only considers the model selection and not whether the actual predictions are in good agreement. The GDT1 was also converted into a Z-score for each target by subtracting the average GDT1 and dividing by the standard deviation.

## Competing interests

The authors declare that they have no competing interests.

## Authors’ contributions

BW conceived the idea and prepared the data. AR and BW parameterized and optimized the method. AR, EL and BW did the analysis and wrote the paper. All authors read and approved the final manuscript.
